# Society Meetings

**Published:** 1902-01

**Authors:** 


### SOCIETY MEETINGS.

THE Medical Society of the State of New York will hold its ninety-sixth annual meeting at Albany, Tuesday, Wednesday and Thursday, January 28, 29 and 30, 1902, under the presidency of Dr. Henry L. Elsner, of Syracuse. The scientific part of the program is being prepared by the business committee, which consists of Drs. Nathan Jacobson, chairman, 430 S. Salina Street, Syracuse; George R. Fowler, Brooklyn, and William C. Krauss, Buffalo. Members and delegates desiring to read papers are requested to communicate with the chairman of this committee.

A rebate will be given by the railroads to members and delegates attending this meeting, but it is necessary in order to obtain this rebate, that a certificate be obtained from the ticket agent at the starting point, which will be given to the purchaser upon request.

---

THE Western Surgical and Gynecological Association held its eleventh annual meeting at Chicago, December 18 and 19, 1901. A large and interesting program had been prepared and the meeting was well attended and took rank as one of the best in its history. The officers were: President, A. F. Jonas, Omaha, Neb; first vice-president, A. W. Abbott, Minneapolis, Minn.; second vice-president, C. E. Ruth, Keokuk, Iowa; secretary-treasurer, George H. Simmons, Chicago, Ill. Executive council: O. Beverly Campbell, Chicago, Ill., Chairman; H. C. Crowell,

Kansas City, Mo., John P. Lord, Omaha, Neb., Jas. E. Moore, Minneapolis, Minn., M. L. Harris, Chicago, Ill. Committee of Arrangements, John B. Murphy, Chairman; A. H. Ferguson, J. Clarence Webster.

---

THE Buffalo Academy of Medicine held meetings during the month of December, 1091, as follows:

*Section on Surgery.*—Tuesday evening, December 3. Program: The division of the sensory root of the trigeminus for the relief of tic douloureux; an experimental, pathological and clinical study, with a preliminary report of one surgically successful case, Charles H. Frazier, Philadelphia, Pa.

*Section on Medicine.*—Tuesday, December 10. Program: Some new ideas in management of typhoid fever, D. W. Harrington; Little's disease, Irving M. Snow; Friedreich's ataxia, Dewitt H. Sherman; A case of microcephalus, L. Schroeter; Is it syphilis of the nervous system? Sidney A. Dunham. A case of congenital idiocy, Julius Ullman.

*Section on Pathology.*—The meeting of this section announced for the evening of December 17, was postponed until further notice.

*Section on Obstetrics.*—Tuesday evening, December 24. Program: Peritoneal tuberculosis, William H. Mansperger; Concerning the management of labor cases, Stephen Y. Howell.

---

THE Esculapian Club, of Buffalo, will hold meetings for 1902, as follows: January, host, H. S. Townsend, essayist, A. J. Colton; February, host, F. W. McGuire, essayist, C. J. Reynolds; March, host, F. J. Carr, essayist, C. A. Clements; April, host, C. J. Reynolds, essayist, C. E. Congdon. The officers are: President, A. W. Bayliss; vice-president, W. B. Saltsman; secretary-treasurer, C. J. Reynolds. The meetings are held on the third Thursday of each month.

---

THE Medical Society of the County of Chautauqua held its semi-annual meeting at Jamestown, Tuesday, December 17, 1901, under the presidency of Dr. N. E. Beardsley, of Jamestown. The program prepared by the secretary, Dr. C. A. Ellis, of Sherman, was as follows: Appendicitis, A. Wilson Dods, Fredonia; (*a*) Serious heart lesions without continuous physical signs; (*b*) Arteriosclerosis in early and middle-life, Henry L. Elsner, Syracuse, President Medical Society of the State of New York; Some complications of heart disease, A. D. Lake, Gowanda; renal and vesical tuberculosis, J. Henry Dowd, Buffalo; Value of ophthalmic examinations in general diagnosis, Geo. E. Blackham, Dunkirk.

